# 3D Pathology Volumetric Technique: A Method for Calculating Breast Tumour Volume from Whole-Mount Serial Section Images

**DOI:** 10.1155/2012/691205

**Published:** 2012-12-23

**Authors:** G. M. Clarke, M. Murray, C. M. B. Holloway, K. Liu, J. T. Zubovits, M. J. Yaffe

**Affiliations:** ^1^Physical Sciences Platform, Sunnybrook Research Institute, Room C7-27c, 2075 Bayview Avenue, Toronto, ON, Canada M4N 3M5; ^2^Physical Sciences Platform, Sunnybrook Research Institute, Room C7-48a, 2075 Bayview Avenue, Toronto, ON, Canada M4N 3M5; ^3^Department of Surgery, Faculty of Medicine, University of Toronto, ON, Canada M5S 7A8; ^4^Department of Surgery, Sunnybrook Health Sciences Centre, Room T2-015, 2075 Bayview Avenue, Toronto, ON, Canada M4N 3M5; ^5^Physical Sciences Platform, Sunnybrook Research Institute, Room C7-27a, 2075 Bayview Avenue, Toronto, ON, Canada M4N 3M5; ^6^Department of Laboratory Medicine and Pathobiology, Faculty of Medicine, University of Toronto, ON, Canada M5S 7A8; ^7^Department of Pathology, The Scarborough Hospital, 3030 Birchmount Road, Toronto, ON, Canada M1W 3W3; ^8^Departments of Medical Biophysics and Medical Imaging, Faculty of Medicine, University of Toronto, ON, Canada M5S 7A8; ^9^Physical Sciences Platform, Sunnybrook Research Institute, Room S6-57, 2075 Bayview Avenue, Toronto, ON, Canada M4N 3M5

## Abstract

Tumour size, most commonly measured by maximum linear extent, remains a strong predictor of survival in breast cancer. Tumour volume, proportional to the number of tumour cells, may be a more accurate surrogate for size. We describe a novel “3D pathology volumetric technique” for lumpectomies and compare it with 2D measurements. Volume renderings and total tumour volume are computed from digitized whole-mount serial sections using custom software tools. Results are presented for two lumpectomy specimens selected for tumour features which may challenge accurate measurement of tumour burden with conventional, sampling-based pathology: (1) an infiltrative pattern admixed with normal breast elements; (2) a localized invasive mass separated from the *in situ* component by benign tissue. Spatial relationships between key features (tumour foci, close or involved margins) are clearly visualized in volume renderings. Invasive tumour burden can be underestimated using conventional pathology, compared to the volumetric technique (infiltrative pattern: 30% underestimation; localized mass: 3% underestimation for invasive tumour, 44% for in situ component). Tumour volume approximated from 2D measurements (i.e., maximum linear extent), assuming elliptical geometry, was seen to overestimate volume compared to the 3D volumetric calculation (by a factor of 7x for the infiltrative pattern; 1.5x for the localized invasive mass).

## 1. Introduction

Tumour size is a commonly used predictor of survival in breast cancer and correlates strongly with lymph node involvement [1–5]. Tumour size is included in the American Joint Committee on Cancer/Union for International Cancer Control (AJCC/UICC) Cancer Staging Manual [6] and is represented by the maximum linear extent of disease.

Here we describe a novel methodology for measuring tumour volume in lumpectomies, referred to as the 3D pathology volumetric technique. For proof of concept, we demonstrate the technique using two lumpectomy specimens, comparing volumes as measured from serial whole-mount sections versus simulated conventional, sampling-based pathology. Serial, whole-mount sections are produced utilizing “3D pathology” techniques and then digitized [7]. Conformational distortion is minimized by first encapsulating the fresh tissue sample in a buoyant, density-matching gel. Automatic microwave processing is employed to accelerate processing of the large tissue samples. Quantitative analysis is performed on the large image dataset (30–70 GB) using custom software tools to create volume renderings and estimate volumes of both* in situ* and invasive disease.

Tumour volume may provide a more accurate representation of size, because tumour is a 3D entity. Volume can vary significantly among tumours which have the same maximum linear extent. A simple ellipsoid model has been used to approximate volume using maximum linear extent and has been shown to provide a more accurate assessment of the volume of breast tumours compared to modelling the tumour as a sphere; in a retrospective study of 165 tumours measuring 2.5 cm or less, the largest diameters in anterior-posterior (AP), medial-lateral (ML), and superior-inferior (SI) dimensions were distinct in 96.4% of the cases [8]. Biologically, tumour volume is proportional to the number of cells, and theoretical models have shown that metastatic potential depends on the total number of cells and the probability of each to disseminate [9]. 

To date there are no clear data supporting the superiority of tumour volume over maximum linear dimension as a predictor of outcome in breast cancer, although current pathology guidelines are beginning to incorporate 3D parameters (e.g., eccentricity factor) [10–12]. Some studies (limited to unifocal tumours) fail to demonstrate stronger prediction with tumour volume, when estimated using the ellipsoid approximation, compared to maximum linear extent [13]. However, for lung cancer, a significant association between tumour volume and both overall survival and disease-free survival has been shown [14, 15]. In staging of prostate cancer, tumour volume has been shown to be a strong predictor of lymph node metastasis [16]. Typically, in conventional work for these sites volume is estimated from linear measurements assuming ellipsoid geometry.

One of the impediments to establishing the prognostic value of tumour volume in breast cancer staging stems from inconsistencies in measurement technique, especially for more complex tumour patterns (e.g., diffusely infiltrating or multifocal). Diffusely infiltrating tumours exhibit a morphology in which the cancer cells are interspersed with normal epithelial and stromal elements making precise measurement of the volume of tissue occupied by tumour cells difficult. Multifocality occurs in about 30% of breast cancers [17] and is associated with local recurrence [18] and decreased survival [19, 20]. Multifocality is also an independent prognostic factor for local relapse and distant metastases [21]. AJCC/UICC guidelines are based on the size of largest focus while some studies demonstrate prognostic value for the aggregate diameter instead [22] or show that volume must be controlled to demonstrate the association between multifocality and lymph node involvement [23]. For multifocal prostate cancer, significant overestimation of mean volume has been shown and at least one measurable tumour is missed in approximately 17% of cases, when the ellipsoidal method is used, assuming a gold standard based on serial sectioning [24]. Similar observations have been noted for lung carcinoma, when estimating volume using an ellipsoidal approximation along with maximum extent measured from serial standard-format histological sections [25].

Whole-mount sections have been proposed as a “gold standard” for evaluating multifocality (defined here as two or more foci of either invasive or *in situ* carcinoma where the foci are separated by intervening normal breast tissue) and may permit more accurate assessment of tumour burden where conventional sampling is difficult (e.g., due to lack of desmoplastic reaction or infiltrative growth pattern). Studies using whole-mount or large-section pathology techniques confirm accepted prevalence rates for multifocality (observed in 31.9% of 1–14 mm invasive breast carcinomas, in a study of 301 consecutive cases) and confirm that multifocality is an independent prognostic factor for survival at 10 years [26, 27]. Furthermore, multifocality is associated with a more than twofold increased risk of vascular invasion and lymph node metastasis compared to unifocal cancer [26]. However, the AJCC/UICC guidelines require only measurement of the largest tumour focus [6]. Whole-mount sections would enable the entire tumour burden comprising any secondary foci to be more fully assessed. 

In this work, we extend the principle of increasing coverage by incorporating serial sectioning, while supporting the flaccid specimen to reduce conformational distortion, to create a 3D representation of tumour histology. We describe a “3D pathology volumetric” technique which utilizes a set of whole-specimen, whole-mount serial section images to create volume renderings and calculate invasive and *in situ* tumour volumes. The volumetric analysis is demonstrated using two lumpectomies with features that might be associated with underestimation of tumour burden when conventional histological sampling is used. Calculated volumes are compared with those obtained from simulated conventional histological sampling, and also with estimated volumes obtained from linear measurements assuming ellipsoid tumour geometry.

## 2. Methods

Two lumpectomy cases were selected retrospectively from the whole-mount tumour bank at the Biomarker Imaging Research Laboratory at Sunnybrook Health Sciences Centre. The specimens were initially obtained from the Department of Pathology at Sunnybrook with the approval of the institutional Research Ethics Board, excluding lumpectomies that would be submitted *in toto *when conventional sampling-based techniques would be used. The diagnosis in both cases was reported as infiltrating ductal carcinoma not otherwise specified (IDC NOS). One case (Case A) is an invasive tumour that infiltrates diffusely, mostly without a desmoplastic reaction or destroying intervening benign breast elements, such that a typical tumour section shows invasive carcinoma admixed with benign breast elements. The other case (Case B) is a localized invasive tumour with foci of *in situ* carcinoma away from the invasive tumour and separated from it by intervening benign tissue. Case B is, therefore, representative of the challenge inherent in measuring tumour volume for multifocal disease, using conventional techniques. 

Both specimens were prepared and processed using techniques collectively referred to as “3D pathology” [7, 28]. Each fresh, unfixed specimen was first suspended in a buoyant gel (3.5% agar), and after setting, each tissue-gel block was serially sliced (in the ML dimension) into uniform, 4 mm thick slices using a rotary slicer (Berkel Products Co., Ltd.; ITW; Glen Lake, Il, USA). The tissue-gel slices were fixed overnight in 10% neutral buffered formalin and then processed using a 16-hour program developed for whole-mount breast tissues in an automatic tissue processor that uses microwave assistance (Pathos Classic; Milestone Medical srl; Sorisole, Italy). The processed slices were embedded in custom moulds and one 4 *μ*m thick tissue section was obtained from the top of each block using a sliding microtome (SM2500; Leica Microsystems, Germany). The sections were stained with hematoxylin and eosin (H&E) using manual techniques. 

**Figure 1:**
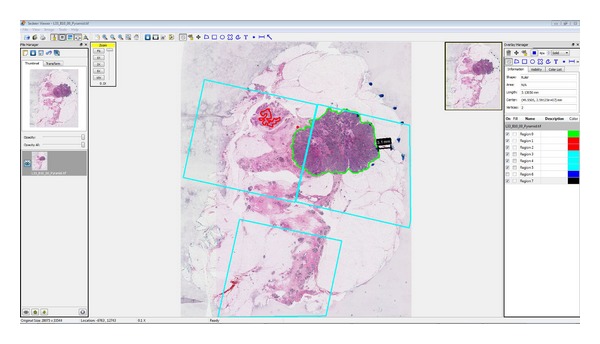
Sedeen viewing program. A whole-mount breast section is annotated, regions of interest are defined, and coordinates are stored for quantitative analysis.

The two sets of whole-mount sections, mounted on 7.62 cm × 10.16 cm glass microscope slides, were produced comprising 14 slides from Case A, and 23 from Case B. The set was digitized (Tissue Scope; Huron Technologies Inc.; Waterloo, ON, Canada) using a pixel spacing of 2 *μ*m as previously determined to optimize tumour detectability and computational feasibility [29]. The size of the total image dataset was 28 GB and 69 GB for Case A and B, respectively (average 0.4–3 GB per section). The images were interpreted by a pathologist (KL) using Sedeen Viewing Program (SElective Decoding and Encoding Engine [30]; developed by Dr. Anne Martel and Danoush Hosseinzadeh at Sunnybrook Research Institute). This software tool was developed to enable interactive display and quantitative work for large image datasets using conventional workstations. Features include panning and zooming, tools for contouring, and annotating and measuring tumours or multiple regions of interest (e.g., invasive tumour and *in situ* tumour) (Figure 1). Using this software, manual, digital contouring of tumour in all of the images was performed and coordinates were stored to file in “extensible markup language” (.xml) format. Using a “virtual sampling” technique described elsewhere [7], a set of images simulating the conventional, sampling-based pathology evaluation (i.e., standard sized slides) was created.

For volume calculations and visualization, the  .xml files were imported into MATLAB (MATLAB7.11.0.584 (R2010b); Math Works Inc.; Natick, MA, USA), to generate a volume rendering for each case, and to calculate tumour volumes, for *in situ* and invasive disease separately. For each whole-mount image, three binary image arrays were generated to represent the following three features as defined by the digital contouring: *in situ* disease, invasive disease, and normal tissue, with pixels enclosed in a contour set to intensity values R, G, B = 1, and 0 otherwise. The three binary arrays were stacked and then extruded in the medial-lateral dimensions, by layering duplicate copies of the arrays at every 2 *μ*m, corresponding to the lateral resolution, to fill the 4 mm gap between whole-mount sections. In this way, isotropic 4 mm voxels were preserved in the volume rendering. Tumour volumes were calculated by Riemann summation of all the positive pixels (RGB intensity = 1) in the volume rendering, for *in situ* and invasive disease separately. For the set of images simulating conventional pathology technique, volume was calculated similarly by multiplying the sampled tumour area by the thickness of 4 mm. 

Finally, tumour volume was estimated from 2D measurements of maximum linear extent in the ML, AP, and SI dimensions using the following ellipsoidal approximation: Volume_ellipsoid_ = 1/6*π* ML*·*AP*·*SI. The maximum linear extent in ML, AP, and SI dimensions was measured from the whole-mount sections.

## 3. Results

**Figure 2:**
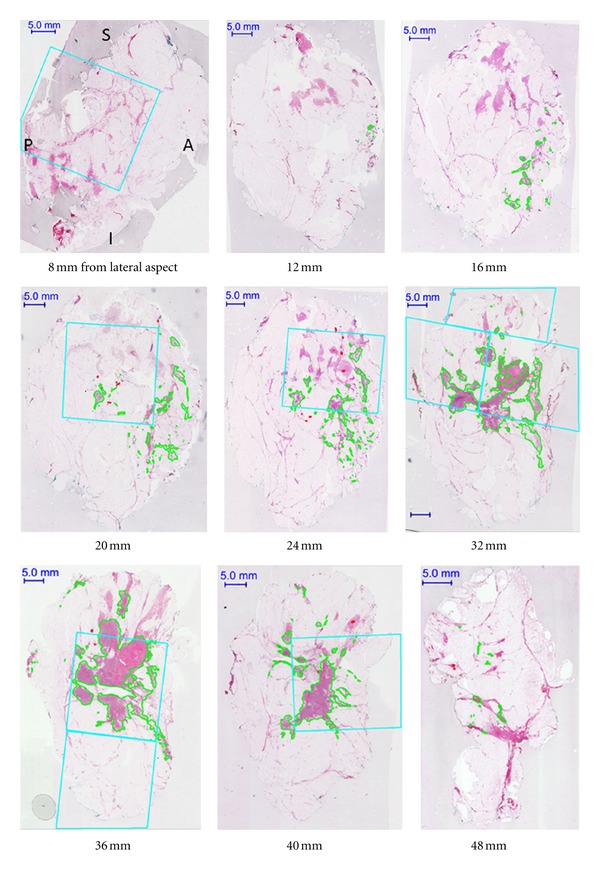
Selected serial whole-mount sections (Case A) in IDC NOS, diffuse pattern. The orientation is consistent for all of the images (A = anterior; P = posterior; S = superior; I = inferior). Invasive tumour is digitally contoured in green. The cyan boxes represent areas which would be sampled in the conventional pathology evaluation, as assessed by the pathology assistant on optical images aided by palpation of tissue slices. There are 14 whole-mount sections in total.

**Figure 3:**
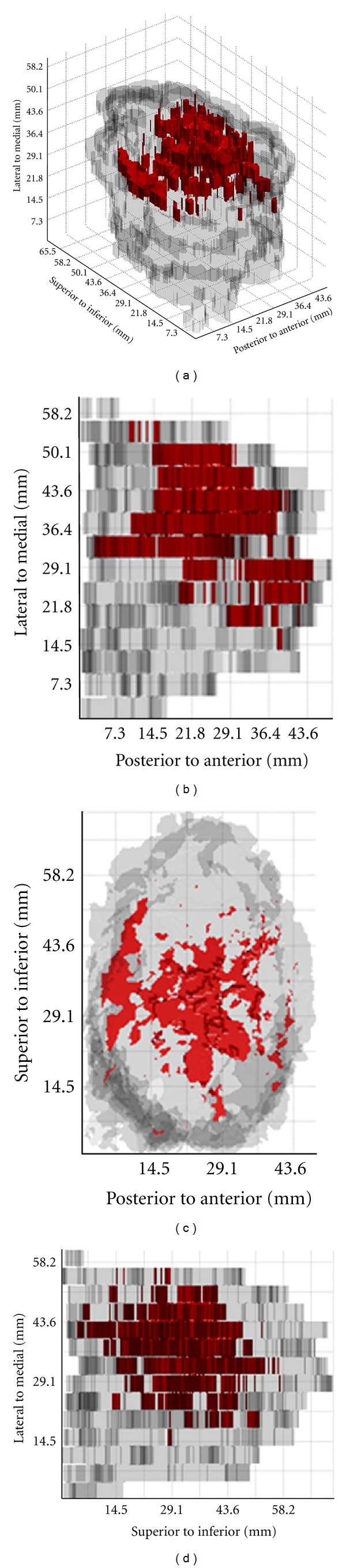
3D tumour visualization for Case A: (a) volume rendering and projection views through the following dimensions: (a) SI, (b) ML, and (c) AP.

**Figure 4:**
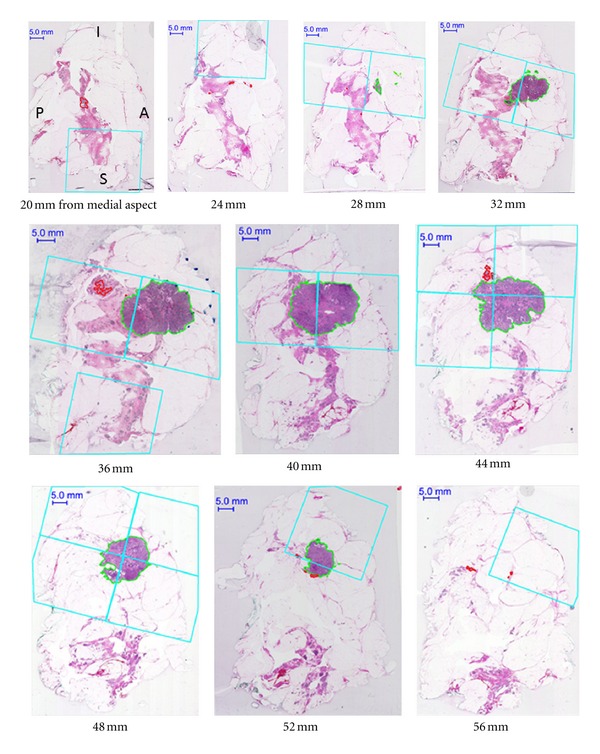
Selected serial whole-mount sections (Case B) in IDC NOS, localized pattern with *in situ* component extending away from the tumour. The orientation is consistent for all of the images (A = anterior; P = posterior; S = superior; I = inferior). Invasive tumour is contoured in green, *in situ* in red. The cyan boxes represent areas which would be sampled in the conventional pathology evaluation, as assessed by the pathology assistant on optical images aided by palpation of tissue slices. There are 23 whole-mount sections in total for this case.

**Figure 5:**
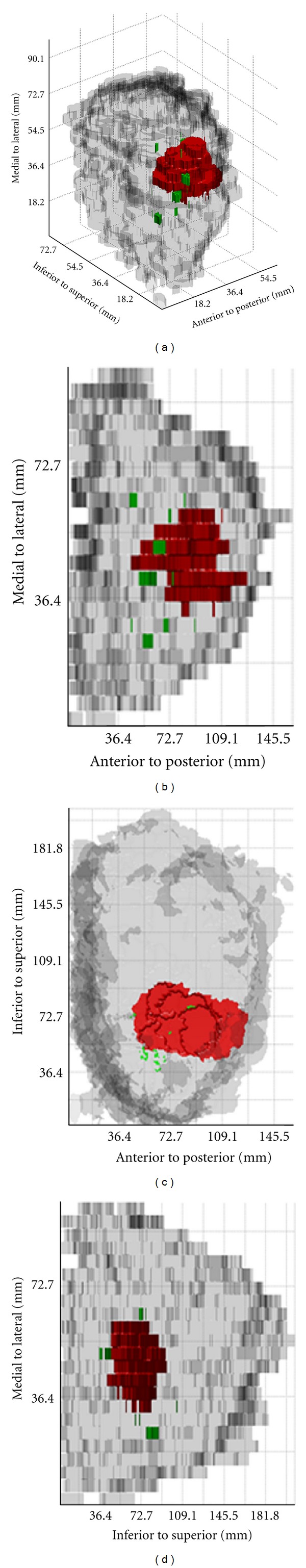
3D tumour visualization for Case B: (a) volume rendering and projection views through the following dimensions: (a) SI, (b) ML, and (c) AP. Regions of invasive tumour are shown in red, and *in situ* disease in green.

For Case A, the set of serial whole-mount sections which contain tumour is shown in Figure 2. These images also depict the locations where conventional samples would be taken as determined by the pathology assistant [7]. The volume rendering for this case is shown in Figure 3. Figures 4 and 5 present the serial whole-mount sections and volume rendering for Case B.

Tumour volume, calculated from the full renderings as well as from the locations corresponding to conventional sampling, are compared in Table 1, along with measurements of maximum linear extent and the corresponding ellipsoidal volume. 

## 4. Discussion

The 3D pathology volumetric technique facilitates visualization of spatial relationships within lumpectomies and may provide a more accurate surrogate for tumour burden. It is seen from Case A that when the tumour infiltrates without eliciting a desmoplastic reaction and without destroying the normal benign breast elements, the conventional 2D measurements using maximum extent in the three dimensions (AP, ML, and SI) may overestimate tumour burden. If the tumour is approximated by an ellipsoid, then using these three measurements the tumour volume is overestimated by a factor of approximately 7 (Table 1). However, when the tumour is localized (Case B), the estimated volume based on measurements of maximum extent assuming ellipsoid tumour distribution more closely approximates the calculated volume, overestimating by a factor of 1.5. Thus, the 3D volumetric technique may remove bias introduced by assumptions of tumour geometry when less precise approximations are used.

From the volume renderings (Figures 3 and 5), the relationship of invasive tumour to the *in situ* component that is present away from the tumour can be readily appreciated. Similarly, the locations of close or involved margins can be easily identified. Taking the close or involved margins seen in the serial whole-mount section images (Figures 2 and 4) as a tissue landmark, corresponding areas are seen in the volume renderings (Figures 3 and 5) within the context of the 3D specimen. For example, tumour at the inferior margin in the section 10 mm from the lateral aspect in Case A (Figure 2) is seen in the volume rendering (Figure 3), relative to another close margin near the inferior-posterior midpoint. 

Even within the limitations of a 2D presentation, whole-mount serial sections can enable more accurate estimates of tumour burden (Case A, Figures 2 and 4) compared to conventional sampling. Case A indicates the power of the 3D volumetric technique to identify and measure the extent of tumour in those lesions in which the tumour cells extensively infiltrate the normal tissue. Comparing tumour volume to the subset which would be sampled, consequently in this case, only 70% of the total invasive tumour volume would have been captured in the conventional approach. For the localized mass in Case B, however, the representation increases to 97%. 

Case B illustrates the ability of the 3D pathology volumetric technique to enhance visualization and resolve tumour foci which are separated by normal tissue. At least two distinct foci of *in situ* disease appear as follows: one at 20–28 mm from the medial aspect and one at 36–44 mm from the medial aspect. In this case, only 56% of the *in situ* component would be captured using conventional sampling. Whole-mount serial sections may also help to capture DCIS, which might otherwise be difficult to sample adequately. Moreover, the focality of the *in situ* component in the ML dimension is better appreciated from the volume rendering (Figure 5) compared to the serial section presentation (Figure 4) in which continuity between serial sections is more difficult to synthesize.

Studies comparing methodologies for measuring tumour volume in other cancer sites support our observations. For prostatectomies, where the majority of tumours assume a multifocal distribution, five techniques were compared. These include the ellipsoidal approximation and Riemann summation for serial sections 3 mm and 6 mm apart. Taking the calculation for serial sections 3 mm apart as the “gold standard,” using the sections 6 mm apart instead would cause the mean tumour volume to be overestimated by 29.5%. Using the ellipsoidal approximation, volume varied widely compared to the gold standard (0.5%–132%) [24]. Similarly for lung tumours, it was shown that Riemann summation (using sections 10 mm apart) is most accurate compared to the ellipsoid approximation and especially compared to a spherical approximation, with the less accurate methods overestimating volume [25]. 

The 3D pathology volumetric technique is well suited to validation of noninvasive imaging modalities (e.g., magnetic resonance imaging) which are used to estimate 3D tumour descriptors including volume and surface area. Surface area might also serve as a useful reporter for tumour burden in conjunction with volume. Our methodology lends itself to exploration of other quantitative measures including surface area as a simple extension of the volumetric principle. Using the techniques we describe here, we can obtain precise, volumetric measurements of challenging features (e.g., separate foci of disease, infiltrating pattern) and in future studies the prognostic significance of these presentations can be explored using the volumetric technique. Studies on the prognostic significance of tumour volume with relation to biology are scarce. It has been suggested that tumour volume is inversely related with microvessel density, and that metastasis may, therefore, be an early event [31]. The 3D volumetric technique can provide a platform for precise, volumetric measurements of both morphological and biological patterns, which can be correlated to validate prognostically significant relationships.

## 5. Conclusion

Using the 3D pathology volumetric technique, tumours are visualized in 3D and spatial relationships between key features (e.g., close or involved margins) are readily appreciated. The technique also enables calculation of tumour volume, which may be a more accurate representation of tumour burden or size compared to 2D linear measurements. This technique also reduces the errors associated with undersampling in conventional histopathology, which can result in underestimation of tumour burden. The volumetric technique may be particularly useful in cases where accurate representation using conventional tissue samples may be difficult (e.g., infiltration without desmoplastic reaction and tumour admixed with normal epithelium, or localized invasive mass with multifocal *in situ* carcinoma away from the tumour). We have shown that in such cases tumour burden can be significantly underestimated using conventional methods. Conversely, tumour volume can be markedly overestimated, especially for more complex tumour distribution (e.g., infiltrative), when simple approximations such as an ellipsoid model are used instead of the volumetric approach. Additional studies to evaluate the predictive value of tumour volume on patient outcome are underway.

## Figures and Tables

**Table 1:** Summary of tumour volumes. Tumour volumes were calculated following three methods: from digitally contoured, serial whole mount sections following the 3D pathology volumetric technique; from the regions indicated by the locations of simulated conventional sampling; from three linear measurements of maximum tumour extent (in ML, AP, and SI dimensions) assuming an ellipsoidal tumour shape.

	Method	Case A	Case B
	3D pathology volumetric	4.179 cm^3^	5.105 cm^3^
Invasive	Conventional samples	2.857 cm^3^	4.954 cm^3^
	Ellipsoidal approximation	29.489 cm^3^ (44 mm × 40 mm × 32 mm)	7.540 cm^3^ (25 mm × 24 mm × 24 mm)

	3D pathology volumetric	N/A	132 mm^3^
*In situ *	Conventional samples	N/A	75 mm^3^
	Ellipsoidal approximation	N/A	6 mm^3^ (2 mm × 2 mm × 3 mm )
